# Caregiver determinants and capacity for participation in constraint-induced movement therapy

**DOI:** 10.3389/fped.2025.1487781

**Published:** 2025-02-20

**Authors:** Hunter G. Moore, Amy Ayala, Bhooma R. Aravamuthan, Alyssa E. Smith, Sharon L. Ramey, Catherine R. Hoyt

**Affiliations:** ^1^ Program in Occupation Therapy, Washington University School of Medicine, St. Louis, MO, United States; ^2^ Biostatistics and Qualitative Research Shared Resource, Washington University School of Medicine, St. Louis, MO, United States; ^3^ Department of Neurology, Washington University School of Medicine, St. Louis, MO, United States; ^4^ Department of Pediatrics, Washington University School of Medicine, St. Louis, MO, United States; ^5^ Fralin Biomedical Research Institute, Virginia Tech, Roanoke, VA, United States

**Keywords:** CIMT, qualitative, EPIs, caregiver-led CIMT, barriers

## Abstract

**Aim:**

Hemiplegic cerebral palsy affects 1 in every 1,100 children, making it the most common pediatric motor disability. Constraint-Induced Movement Therapy (CIMT) is an evidence-based intervention that significantly improves upper extremity function when implemented with high fidelity. Despite its effectiveness, CIMT's intensive nature—requiring daily therapy for up to twenty days—limits its availability. This study examined caregivers’ perspectives on implementing and adapting home-based CIMT to identify practical solutions for improving intervention accessibility.

**Method:**

Caregivers of a child who has a diagnosis associated with upper extremity motor impairment consistent with cerebral palsy were recruited from the Cerebral Palsy Center at the St. Louis Children's Hospital. Caregivers completed a semi-structured interview to share their CIMT experiences, as well as their ideas and opinions related to modified versions of CIMT. All interviews were coded and analyzed for themes using descriptive analysis.

**Results:**

Twelve interviews were conducted and revealed that caregivers would be interested in CIMT with an at-home model. Those who had experience with CIMT stated they found meaningful results from their participation in CIMT. Caregivers communicated potential challenges such as their child remaining engaged in at-home therapy, caregiver confidence in implementing the therapy, and the time required for implementing caregiver-led, home-based CIMT.

**Interpretation:**

Study findings identified that caregivers see value in a modified, at-home CIMT program. Developing a modified version of CIMT is needed to increase access to this beneficial intervention.

## 1 Introduction

Cerebral palsy (CP) is a group of neurological disorders that appear in infancy or early childhood that permanently affect body movement and muscle coordination (1) CP is most commonly caused by pediatric stroke (PS), which affects approximately 1 in 1,100 children (2). Children with CP secondary to PS experience lifelong morbidities including physical disabilities, cognitive and communication disorders, behavioral and mental health challenges, and epilepsy (2–6). CP can result in an increased economic burden (7, 8) on caregivers through more frequent hospitalization, longer hospital admissions, and other healthcare needs (9). CP can also affect children's daily routines, developmental progress, quality of life, and societal participation (4, 10). Providing the best-known interventions at the earliest opportunity may help children with cerebral palsy reach their potential, by maximizing the early plasticity of the developing brain (10–13). Constraint-Induced Movement Therapy (CIMT) has historically been provided to children with upper motor impairment consistent with CP and PS.

CIMT is one of the most studied evidence-based interventions. When implemented with high fidelity, it can result in improved long-term functional upper extremity movement in children with high satisfaction levels (14, 15). CIMT is an intervention that has been identified to improve motor outcomes in children with hemiplegic cerebral palsy, often caused by stroke (3, 14, 15). In published models, CIMT involves restraining the less affected upper extremity for most of the day (90% or more), in which the affected upper limb is required to perform everyday activities and engage in specialized therapy (15, 16). However, CIMT treatment protocols vary widely by dosage (intensity of therapy and duration) and constraint type (e.g., cast, mitten, sling) (15, 16). Further, one qualitative survey conducted in the United States with occupational and physical therapists on CIMT found that 75% would have difficulty administering CIMT in their clinics, and 83% felt that most clinics lacked adequate resources to implement CIMT (17). The participants in this study reported that the length of time wearing the constraint, the number of hours required to participate, and the likelihood of insurance covering CIMT services were some of the biggest barriers (17). A quantitative study found three key findings in CIMT implementation; there is a notable gap in the availability of pediatric CIMT programs and access, programs vary greatly (length of program, hours of therapy, constraint used), and there is limited coverage of pediatric CIMT in rehabilitation education (18). The intensive nature of CIMT, requiring 3–6 h of direct therapy daily for 17–20 days, limits its accessibility for many eligible children (19). Provider concerns about intervention intensity, cast usage, insurance reimbursements, and scheduling difficulties further restrict CIMT's availability for those who could benefit the most from it (19). Few studies have explored caregiver perceptions of CIMT, and to our knowledge, none have explored caregiver-driven perceptions and suggestions for developing a model of CIMT that could be more easily implemented for children with upper extremity motor disabilities (20, 21).

Parental involvement in pediatric rehabilitation is essential, as caregivers are responsible for designing opportunities for their children. Caregivers play a critical role in creating a consistent environment for therapy by integrating activities into daily routines. Actively engaging with families in rehabilitation, which includes developing goals and objectives is central to successful therapeutic interventions (22–25). Engaging with families in setting goals that align with the child's needs and family's daily life is important, making the goals more effective and practical. There have been a few qualitative studies that examined determinants in parent-delivered therapies for children with or at risk for CP, with parent involvement being highlighted as essential (26, 27). Fostering parent-delivered interventions requires addressing gaps in knowledge, skills, and building the capacity for parents and therapists together (26, 27). Building a relationship and enabling the parents to see an intervention as a priority is important in delivering the therapy (27). Family-centered interventions, with targeted goals, like a home-based CIMT model, could improve outcomes for children with upper extremity motor disabilities. A recent publication emphasized the advantage of having an intervention program (Small Step) at home with coaching and flexible support from a team of therapists (25). They identified an enhanced capacity to provide an enriching opportunity in everyday life but recognized the potential challenges and barriers in addressing individual family needs.

The purpose of this study was to identify key factors, including facilitators and barriers to caregiver and family participation in CIMT interventions to uncover strategies to increase the future implementation of CIMT for children with a diagnosis associated with upper extremity motor impairment consistent with CP.

## 2 Methods

Approval was obtained from the Institutional Review Board at Washington University School of Medicine. The study employed a qualitative, cross-sectional design, utilizing semi-structured interviews with caregivers of children with a diagnosis of associated with upper extremity motor impairment consistent with CP. The research team had clinical, personal, and research experience in qualitative methods and CIMT. Informed consent was obtained from all participants before inclusion in the study.

The Exploration, Preparation, Implementation, Sustainment (EPIS) framework was used to gain a comprehensive understanding of the contextual factors influencing the implementation of CIMT in real-world settings (28, 29). This study primarily focused on the Exploration phase to identify caregiver-driven determinants of CIMT participation, and to develop a modified version of CIMT based on caregiver-identified priorities. The inner and outer contexts from the EPIS framework informed the development of interview questions aimed at understanding the factors limiting CIMT accessibility for families. The study adhered to the Consolidated Criteria for Reporting Qualitative Research Checklist (COREQ) guidelines to ensure rigorous and transparent reporting of the research process and findings (30).

### 2.1 Participants

A purposive recruitment strategy was used to recruit caregivers who represent the diversity of populations served in the region (31). Demographic variables including the participant's address, education, ethnic group, household income, and child age were recorded. A recruitment goal of 12 was established to be consistent with qualitative research guidelines (32–34). Caregivers were recruited from the Cerebral Palsy Center at St. Louis Children's Hospital. Eligibility criteria included being the parent or legal guardian of a child aged 2–16 years with a diagnosis associated with upper extremity motor impairment consistent with CP. All children in this study had a diagnosis associated with upper extremity motor impairment consistent with CP of multiple etiologies. While it was originally targeted toward caregivers of younger children (2–5 years of age), recruitment was expanded to include older children to better understand the breadth of caregiver experiences. To ensure representation of a variety of lived experiences, families were approached to participate based on their child's Gross Motor Function Classification System scores (35), the Manual Ability Classification System (36), gender, race, and CP diagnosis. Seven families had a child who previously received CIMT, or some type of constraint was used in therapeutic sessions. Recruitment occurred following a regularly scheduled clinic visit or via phone call. Exclusion criteria included non-consent to recording or non-English speaking caregivers; however, no participants were excluded for these reasons. Caregivers were compensated for their time.

### 2.2 Data collection

Demographic data was collected using the Research Electronic Data Capture (REDCap) service hosted at Washington University (37, 38). The semi-structured interview consisted of 28 semi-structured questions intended to take 45–60 min to complete. A semi-structured interview format allowed for detailed data from fewer participants. The questions were developed by a team of experienced pediatric neurologists (AS, BA), a psychologist (SR), and an occupational therapist (CH) with experience in implementation science and CIMT. The questions were reviewed by a parent council supporting a larger trial on CIMT (39), and feedback was incorporated. Mock interviews were conducted with two parents from the parent council to ensure that questions were clear, kind, and addressed the research question. We acknowledge that our research team lacks the lived experience of caring for a child with cerebral palsy, which may limit our understanding of caregivers’ daily experiences and challenges. Interviews took place through June and July of 2023.

The interview guide provided a brief introduction to CIMT and the purpose of the study. Caregivers were asked about their experiences with CIMT and about their interests and preferences for the potential development of a modified version of CIMT (e.g., home-based and caregiver-led). Table 1 offers a look at how the interview questions are were created utilizing the EPIS construct. The full interview guide is available in the Supplementary Material Appendix and was reviewed by two caregivers of children with a diagnosis of associated with upper extremity motor impairment consistent with CP. Virtual and in-person interviews were offered; however, all participants selected video conferencing for convenience. Interviews were conducted by a pediatric neurologist (AS) or an occupational therapist (CH) over a HIPAA-secure video conferencing platform (Zoom Video Communications). Recordings and transcripts were stored on a secure server.

**Table 1:** Sample interview questions and EPIS construct.

EPIS construct	Sample question
Outer context: patient characteristics	What changes were you expecting (if any) with incorporating this constraint/restraint into therapies/play?
Outer context: patient/client advocacy	If you were to engage in at-home therapies (like CIMT) directed by you, how would you prefer to be trained on how to do these therapies?
Inner context: organizational characteristics	What would be important to you to see in a CIMT program?

### 2.3 Data analysis

The interviews were transcribed from audio recordings using Trint Software (40) and subsequently de-identified to maintain participant confidentiality. Two team members (HM, AA) independently coded the transcripts to ensure intercoder reliability. All transcripts were coded using NVivo 20 (41). The full codebook is available in the Supplementary Material Appendix.

A target recruitment of 12 allowed the research team to achieve a deep understanding of the families’ experiences and allowed for a more in-depth analysis of the interviews. Our participants had a child with a diagnosis associated with an upper extremity motor impairment consistent with CP, which allowed for more meaningful perspectives. As this study is aimed at examining caregivers’ perspectives on implementing and adapting home-based CIMT to identify practical solutions for improving intervention accessibility, a group of 12 participants allowed us to identify themes for future applications.

We employed a descriptive approach in our thematic analysis (42–44). The themes represent the experiences of the caregivers who were interviewed. Descriptive analysis helps capture all the elements of the experiences of the caregivers (42–44). A descriptive analysis was used to examine the caregiver perspectives of CIMT in its natural state. The flexibility of descriptive analysis allowed the themes to focus on the lived experiences of caregivers related to CIMT and ideas for how implementation could be improved to increase the accessibility of the intervention. Common patterns were identified from the interviews. Coders created an initial codebook which was coded by two authors (HM, AA). Throughout the data analysis, the codebook was iteratively refined, with five revisions conducted to ensure alignment with our interview data and methodological rigor. To establish inter-coder reliability, both authors conducted systematic reviews where they independently coded a subset of the same data and then met to discuss and resolve any coding discrepancies, ensuring a consistent interpretative approach. Patterns were grouped into broader themes and were adjusted until all interviews yielded an in-depth understanding of their lived experiences and perspectives of CIMT. This iterative process ensured that the emerging data were deeply rooted in the participants’ experiences.

## 3 Results

Potential participants were identified through the electronic health system and were approached in clinic or over the phone until target recruitment was met. A total of 23 families were approached, and 12 participated in the study. One family declined to participate, and 10 families could not be reached. Demographic information for caregivers and children is summarized in Table 2. Demographic characteristics not endorsed by any participants were not included.

**Table 2:** Demographics of caregivers and children (*n* = 12).

Caregiver demographics	*n*
Gender	
Male	3
Female	9
Race/ethnicity^a^	
White	11
Black or African American	1
Age in years (mean, range)	36.75 (23–62)
Type of community	
Suburban	8
Rural	3
Other	1
Income (mean, range)	$66,439.50 ($30,334-$99,500)
Area deprivation index	
1–3 high-resource area	7
4–7 average-resource area	3
8–10 low-resource area	1
N/A	1
Child Demographics	*n*
Gender	
Male	7
Female	5
Race/ethnicity	
White	9
Black or African American	2
Asian	1
Age in years (mean, range)	6.2 (2–16)
Motor deficit diagnosis	
Unilateral right hemiplegia	5
Unilateral left hemiplegia	5
Bilateral symmetric diplegia	1
Bilateral right predominant triplegia	1
Diagnosis	
Cerebral palsy	10
Other motor disability (stroke, partial paralysis, not formally diagnosed)	2
Prior CIMT intervention	
Yes	7
No	5

CIMT, constraint induced movement therapy.

^a^ No participants reported to identify as Hispanic/Latino.

**Figure 1:**
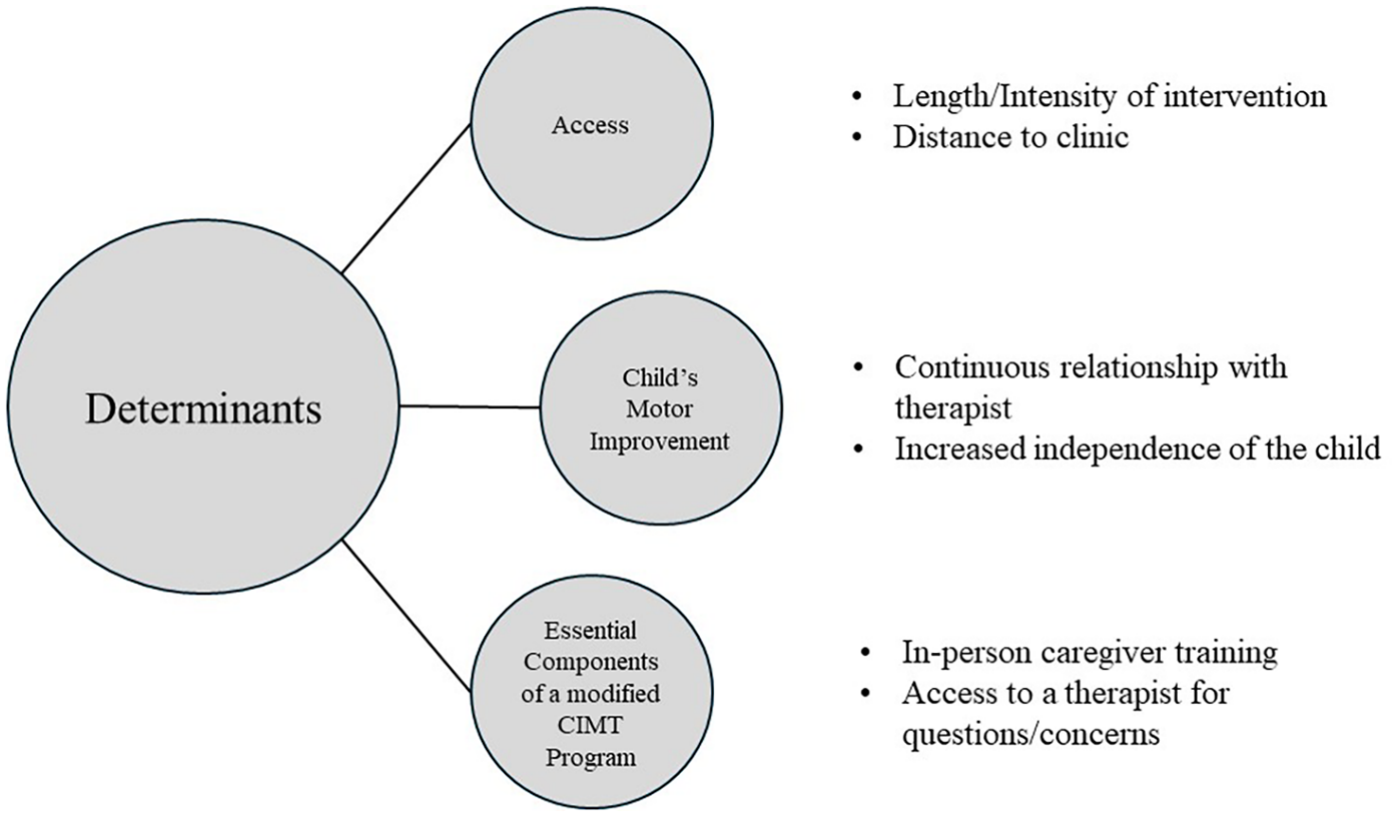
Determinants of caregiver's participation in constraint-induced movement therapy (CIMT).

Interviews lasted an average of 54 min (range: 38–85 min). Initially, nine primary codes were identified from the data. The nine codes were subsequently re-analyzed and refined into eight primary codes and nine secondary codes, resulting in three main themes (Figure 1).

### 3.1 Theme 1: Time and access were primary challenges to participating in CIMT

Caregivers expressed several challenges that hindered their ability to have their child participate in CIMT. These challenges were mainly related to time constraints, distance to therapy locations, and lack of post-therapy guidance. Caregivers emphasized that the time requirement for the intervention, which typically involves 4 weeks of full-day therapy, was difficult to adhere to due to employment or other responsibilities. One caregiver highlighted this challenge, stating:

It is a large amount of time to be doing therapy…because before that, the longest she’d ever done therapy was…60 min, and by…the end of that…she was tired and… it was…a struggle. (Participant 1, previously received CIMT)

The intensive and lengthy nature of CIMT, compared to previous therapies, posed a significant adjustment challenge to caregivers. Towards the end of CIMT sessions, caregivers often struggled to motivate their child, as enthusiasm and engagement waned. The demand to dedicate 3–4 weeks exclusively for therapy was also seen as unrealistic due to other responsibilities. The need for extended leave from work, arranging childcare for other siblings, and putting other commitments on hold was overwhelming:

It was difficult to figure out when was the best time to do it [CIMT], when there’s other kids in the mix and then my work schedule and all the other things you have to do in life. (Participant 9, previously received CIMT)

Additionally, caregivers expressed concerns about the limited accessibility of CIMT, as many families did not reside near clinics offering this specialized therapy. Caregivers noted that traveling to participate in CIMT could be a significant financial challenge and might require taking time off work, securing accommodations, and incurring additional expenses associated with travel and lodging. Caregivers shared their perspectives, stating:

That’s like a weeklong at [therapy site]. But we live in [city], which is almost 3 h away, so it would’ve required us to stay there for a whole week…logistically, it was going to be hard. (Participant 9, previously received CIMT)

And for us, that’s about a 2-hour drive. But it would be a literal Monday through Friday, like we would have to be up there 8 h a day. It was a, like a 3-week course, and honestly, financially, we couldn’t, like there was no way to take off work for that amount of time. (Participant 4, not previously received CIMT)

Caregivers repeatedly emphasized that participating in CIMT would essentially become a full-time commitment and the distance they would need to travel to reach a clinic offering CIMT often surpassed what was manageable. The prospect of dedicating such a substantial amount of time and resources to CIMT left many caregivers feeling overwhelmed to pursue this intervention for their child.

Furthermore, caregivers expressed a strong desire for more support and guidance after completing CIMT. They highlighted the frustration and difficulty they encountered when seeking therapists who were knowledgeable and familiar with CIMT techniques. The lack of access to professionals well-versed in CIMT left caregivers feeling isolated and unsure about how to continue supporting their child's progress. One caregiver shared their experience, stating:

Yeah, it was pretty difficult to find [CIMT]. I think there’s only two [occupational therapists] at all of our Children’s Hospital who had been trained and even knew what this was, so I was kind of shocked by that. (Participant 9, previously received CIMT)

Caregivers communicated pervasive uncertainty about what they should be doing to best help their child. They expressed frustration with a lack of clear guidance and specific instructions for continuing the intervention at home.

I still feel like there’s not really any guidelines…that, you know, you should do X number of minutes, X number of days for X number of weeks. You know, I just feel like I’m winging it all the time. (Participant 1, previously received CIMT)

The primary challenges of CIMT communicated by the caregivers align with the outer context factors of the Exploration phase of EPIS (location and transportation difficulties) and the inner context (caregiver schedules) that could impact participation. Understanding the barriers to participation is an important part of the exploration phase.

### 3.2 Theme 2: Caregivers perceived CIMT to be helpful

Caregivers shared their experiences with and perceptions of CIMT, highlighting both positive aspects and challenges they encountered. Many caregivers expressed enthusiasm for CIMT, noting the encouraging progress they had observed with their child's development, especially in motor function and increased engagement in play and independence.

Caregivers with a child who had received CIMT praised the expertise and support provided by their CIMT team and felt this was an important component in their child’s success.

[CIMT] was awesome. Our therapist was incredible. I have never seen a 2-year-old child be that engaged with one person doing, I mean, they were doing different things, that child was engaged for 6 h straight. It was incredible. (Participant 1, previously received CIMT)

It was interesting. We’ve never done anything like that before, and I really liked it because we saw things that we didn’t think [my child] could do. (Participant 3, previously received CIMT)

Caregivers expressed gratitude and enthusiasm for the opportunity to participate in CIMT with their children. The dedication and perseverance demonstrated by the children were a source of inspiration for the caregivers, who felt encouraged and motivated by their child's achievements. Specifically, caregivers described the excitement of observing their child gain functionality in the affected arm and beginning to recognize the potential of the affected limb, opening new possibilities for growth.

Caregivers felt that improvements in mobility and function boosted the child’s confidence and self-esteem, which also positively affected the entire family, fostering a sense of hope and optimism.

[My child] still chooses to use lefty [left hand] and over any time that [my child] has to choose righty. But now [my child] does know, when [my child is] trying to climb a ladder where like two hands is easier, it’s automatic that [my child] put it [their right hand] up there now. (Participant 8, previously received CIMT)

Theme 2 revealed the perceived value and benefit of CIMT, and the study was able to gauge its potential for adoption of CIMT. Overall, caregivers perceived CIMT to be effective, which is critical in fostering buy-in among families.

### 3.3 Theme 3: Essential components of a home-based CIMT program

Caregivers expressed a high level of interest in participating in a parent-led, home-based CIMT program, and they felt that having the option to do therapy at home would help them learn what they could be doing better to help their child in their natural environment. Caregivers preferred in-person training so they could ask questions and receive feedback before implementing the program at home on their own. Caregivers of children who had received CIMT and those who had not, both preferred having a therapist first but had similar ideas on receiving training to implement a modified session.

I would at least want one in-person session so I could learn from that [physical therapist] or whoever is going to be showing me how to do it. (Participant 2, not previously received CIMT)

That [having a therapist specialized in CIMT] would be ideal, but I would be happy with guidelines. I feel like right now there’s nothing that anyone has given me that says, this is what you should do at home. (Participant 1, previously received CIMT)

Caregivers were eager to have support so that they could provide the best therapy possible for their child. Caregivers desire to still have access to professional guidance from a therapist, which leads to maintaining a relationship between the therapists and the family in supporting their child's development. A caregiver who had not received CIMT highlighted a desire to have consistent check-in to keep on track and continue to provide high-quality therapy for their child.

If there was an in-person, like once a week where [the therapist] could come in and check up on how things are going…kind of give me some ways to continue to push her a little bit harder. (Participant 11, not previously received CIMT)

Of the caregivers interviewed, 9 out of 12 expressed that they would be interested in continuing CIMT on their own at home. Caregivers of children who had received CIMT and those that did not, both expressed that being at home was an advantage, and one caregiver who previously received CIMT would have preferred it to be that way the entire time.

I think that in home is where [my child] would be most comfortable. Obviously, this is where [my child] lives, this is where we do all of [my child’s] therapies. So yeah, that would be a huge advantage of doing it in home. (Participant 2, not previously received CIMT)

I think it really depends on the family, for us it would have been wonderful to do it that way [at-home CIMT]. (Participant 8, previously received CIMT)

Caregivers identified several benefits and described that they would want to participate in CIMT at home, where caregivers would be more involved in providing the intervention. Caregivers described a desire for in-person training prior to starting CIMT. Some caregivers identified that CIMT training “refreshers” would be acceptable in a virtual format, but for starting, in-person is strongly preferred. Caregivers also expressed that completing CIMT at home could help in translating therapy activities and strategies for their child in the natural environment.

### 3.4 Theme 3b: Caregivers’ hesitations about feasibility of home-based CIMT

Caregivers repeatedly expressed interest in leading a home-based CIMT therapy for their child, but several caregivers who had received and who had not received CIMT previously expressed concerns about how it could be feasible. Caregivers identified concerns including the child's ability to remain engaged in their therapy with a parent/caregiver, caregiver confidence in implementing CIMT adequately, and time. Caregivers of a child who had not received CIMT were primarily concerned with not being confident in delivering an intensive therapy effectively. Caregivers of a child who had not received CIMT described that their child may behave differently for them, compared to a therapist, and not engage in therapeutic activities as well.

My only concern would be, not to have that reassurance that I’m doing it correctly, that was always a concern of mine even with [my child’s] other therapies. (Participant 4, not previously received CIMT)

Just getting [my child] to participate at times. I think that would probably be the hardest with me. [My child] is very good about listening to [their] therapists and teachers and things at home, but we have a lot more behaviors than we do anywhere else. (Participant 5, not previously received CIMT)

Caregivers who had previously completed CIMT described the difference between receiving therapy at a facility (e.g., hospital or therapy clinic) compared to home. Caregivers felt that facilities had more resources (e.g., toys, play equipment), which were more engaging for the child over the course of the intensive CIMT intervention.

What I like about going to a facility is that they have different toys and games and different things that you can do. If you don’t have a lot of these activities at home, then the child can become bored and complacent. (Participant 8, previously received CIMT)

Caregivers of a child who had not received CIMT expressed concern about the length of time wearing a cast on the child's dominant arm and how that would affect the family. All caregivers indicated that having a cast on their child at certain times of the year (school time, summer) could limit participation in activities that the child would typically be able to do.

I think my concern would be the length of time that you would be casted. I just, I guess that would be tricky when it comes to logistics, either for school or, you know, if it’s summer break. (Participant 9, previously received CIMT)

While home-based was desirable, caregivers identified barriers to CIMT at home. Many caregivers described feeling nervous about conducting the therapy themselves without a therapist with them. Other barriers included caregivers’ concerns that they would not be able to keep their child's attention for the expected duration, and finding time to dedicate to CIMT daily.

Identifying anticipated facilitators and barriers to an at-home CIMT program is core in the exploration phase of EPIS, with it aligning closely with the inner context. Caregiver readiness, required resources, and capacity are important in understanding the feasibility of CIMT at-home. Identifying what caregivers want and require will help lead to the development a caregiver-driven CIMT model for implementation.

## 4 Discussion

This study aimed to understand the factors influencing caregiver involvement in CIMT interventions, identifying both facilitators and barriers. Three primary themes emerged from the interviews: time constraint and access, positive experiences with CIMT, and supportive needs for successful implementation. Time constraints and access to CIMT were significant barriers to participation, whereas in-person training and ongoing support were identified as crucial facilitators. The themes identified through our interviews correspond with the exploration phase of the EPIS framework, particularly regarding how caregivers navigate early care decisions (28, 29). During the exploration phase, a needs assessment is critical for identifying service gaps and opportunities to improve implementation. Themes from this qualitative study identified specific needs and challenges in delivering upper extremity interventions at home for children with unilateral cerebral palsy. Caregivers identified key facilitators and barriers that will inform the development of a caregiver-led modified CIMT program. Understanding these factors is essential for transitioning to the preparation phase of EPIS and ultimately implementing an effective home-based intervention program. Another outcome from this study is that 9 of 12 families communicated that they would be interested in continuing CIMT on their own at home, despite only 7 of the families having done CIMT in the past. These findings indicate that caregivers understand the value of engaging in high-intensity intervention and want to pursue it for their families but cannot do so presently. Caregivers who had a child who participated in CIMT explained the improvement they saw in their child, which is supported by prior studies (14, 15). Families communicated the importance of providing the best treatment for their child.

Similar to prior work (19) this study found that time and access are primary barriers to participating in CIMT; however, caregivers who did participate in CIMT reported that they wanted more support following their participation in CIMT. More support following their participation in CIMT was desired. For example, caregivers described wanting new activities to do with their child, regular check-ins, and making sure that they were conducting the therapy appropriately. The constraints of time and distance prevented several families from being able to follow through with the therapy. Caregivers expressed that they were unsure about the commitment of CIMT because it was unclear what the potential gains would be for their child. These themes deepen our understanding that families are interested in implementing CIMT at home with adequate support and training.

These findings support previous studies, identifying the important role of caregivers in intervention and rehabilitation (22–25). CIMT is only possible with significant buy-in from those who would be involved, and barriers need to be addressed to help make the intervention a possibility for families that could use CIMT. Caregivers of a child who completed and did not complete CIMT identified that conducting CIMT at home helps address the barrier of distance. Families communicated the importance of being trained and having support in delivering the therapy themselves to ensure their child is receiving the best care available. These results align with prior work (25), where caregivers highlighted the desire to receive coaching at home and being able to integrate the therapy into their everyday routines.

Limitations present in this study included that our cohort was not diverse, as children predominantly identified as White, with three identifying as another race. This is representative of the population in the region (45). We believe the range of socioeconomic statuses contributes to the validity of these results, as cost and accessibility are reported as common barriers (17, 18). Caregivers primarily reported to identify as female, with only three caregivers being male. However, this is representative of who is caring for children with CP more broadly (46, 47).

## 5 Conclusion

To our knowledge, this is one of the first studies to examine caregivers interested in a caregiver-led, at-home version of CIMT. The semi-structured interview questions allowed the caregivers to expand on their lived experiences with CIMT. Caregivers highlighted the difficulties and positive benefits, as well as their interest in participating in a modified program. The next steps include developing and testing implementation strategies of a modified version of CIMT.

## Data Availability

The raw data supporting the conclusions of this article will be made available by the authors, without undue reservation.
