# Supplementary material for: Caregiver determinants and capacity for participation in constraint-induced movement therapy
Source: Front Pediatr. 2025 Feb 20;13:1487781. doi: 10.3389/fped.2025.1487781 (PMC11882512; doi:10.3389/fped.2025.1487781)
Supplement: Supplementary file 1 [file Datasheet1.docx]

**Appendices**

**Appendix A – Finalized Codebook**

| **Code** | **Definition** |
| --- | --- |
| **Therapy** | Any mention of physical therapy, occupational therapy, speech therapy, and so forth. It can include any previous or current therapy that the child has had, as well as any expectations the parents have for their child’s therapy. This code also includes any mention of Botox treatment. This code does not include CIMT (Constraint-Induced Movement Therapy). |
| **CIMT (Constraint-Induced Movement Therapy)** |  |
| - History of CIMT | Any reference to parents’ previous awareness of CIMT or any CIMT that the child has undergone. |
| - Medical advice/Medical team interaction | Any mention of outpatient care the child received. Any reference to previous interactions with clinicians or therapists. |
| **Barriers of CIMT** | Any mention of CIMT barriers, or the lack of. This code includes any challenges they have faced, such as travel, time, or parents’ confidence in providing at-home therapy. |
| **Expectations of CIMT** | Any indication of parents’ or caregivers’ previous or current expectations of CIMT. This includes any reference to awareness/improvement of the affected limb, as well as any description of changes that they observed, whether no change or a significant difference. |
| **Social Support** | Any mention of family/friends, social media support groups, resources, or lack of support. |
| **Ideal Program** |  |
| - Potential challenges | Any mention of potential challenges that the parent or caregiver might face with the potential program. For example, any reference to what they dislike about the potential program. |
| - Program preferences | Any mention of what parents/caregivers want to see in a program. For example, activity logs, home-based therapy, and so on. |
| - Interest in future program | Parent/caregiver mentions if they would or would not be interested in being part of a potential treatment or program. |
| **Family Life** |  |
| - History of diagnosis | Any mention of a child’s medical history, such as when they were diagnosed. |
| - Family activities | Any mention of out-of-the ordinary activities or excursions in which parents/caregivers and their children participate. |
| - Day-to-day life of the child and family | Any mention of their day-to-day or family life. For example, those living in the home, the occupations held by the parents or caregivers, or family routine. |
| - Life skills | Parents/caregivers teach/encourage their child’s basic life skills. For example, bathing, dressing, etc. This code also includes any mention of lack of life skills the child may have. |
| **Parent/Caregiver Confidence** | Any mention of parents or caregivers going out of their way to advocate, seek resources, or figure things out on their own. |

**Appendix B – Interview Guide**

**Introduction**

My name is ____________. The purpose of this discussion is to get your opinions on how we may best develop an at-home therapy guide for a certain type of intervention for people with motor deficits that affect one side of the body, or cerebral palsy. This intervention is called: Constraint-Induced Movement Therapy, or CIMT.

We’ll first learn about your experience with or knowledge of CIMT, then about building the at-home therapy guide, and finally learn more about the person who you care for and your family. We will use the information gained from this interview to develop an intervention program to serve families in the St. Louis region.

This interview should take 45 minutes to 1 hour of your time.

This discussion will be completely confidential, will not be shared with your clinical providers, and will not affect your clinical care. Please know we are trying to get a realistic picture of families’ needs: there are no right or wrong things to say. All of your experiences and opinions are valuable for us to hear.

This information will be shared with our research team. I want to make sure we’re on the same page during this discussion. Please feel free to interrupt me at any time to ask me to clarify or to check in that we’re both talking about the same thing.

To make sure we save all of your responses accurately, we need to record this conversation. Is it OK to begin that recording now?

**History of CIMT/Therapies**

- Do you have anything you’d like to share before we get started?
- Have you heard of CIMT before?
- How would you describe CIMT?
- Have you ever used a constraint/restraint during therapies or play with your child?
- (If they ask for examples) Examples include a sock, a mitt, a splint, or a cast.
- Can you describe what these therapies involved?
- (If needed) Describe a typical therapy or play session involving a constraint/restraint.
- (If needed) Was it therapist directed? Where were the sessions (e.g., clinic/home/both)? How often and how long were sessions with your therapist? How often did you engage in constraint/restraint usage with your therapist present? How about when they weren’t present? For how many weeks or months did you use constraints/restraints during therapies or play?
- What changes were you expecting (if any) with incorporating this constraint/restraint into therapies/play?
- Did you feel like your expectations were met?
- Can you describe your interactions with your therapists and other medical providers regarding use of constraints/restraints?
  - - e.g., How did they support or encourage you? Did you feel any sense of discouragement from them? Was it discussed at all?

**CIMT Explanation**

Here is one explanation of CIMT: CIMT is an established medical intervention designed for people with hemiparesis—where one side of the body is weaker than the other side. It involves casting their stronger arm for a period of at least 3 weeks to encourage the use of their weaker arm. At the same time, the person undergoes in-person therapies for at least 3 hours a day for 5 days a week to strengthen and encourage the use of their weaker arm. The idea behind CIMT is that a young person’s brain, because it is so good at learning new skills, will learn to use the weaker arm better if it is used more. The main goal of CIMT is to improve the functional use of the person’s weaker arm.

- What are your thoughts of CIMT as just described?
- Is CIMT as described an intervention you would pursue for your child now? Why or why not?
- We want to learn more about what you, as a caregiver, are seeking. With this kind of therapy, what would you like to see happen for you, your child, and/or your family?
- (If needing clarification) What do you think could change after this therapy that could be really important for you, your child, or your family?
- (If needed) Can you describe the benefits you think your child might get from CIMT?
- Can you describe why these changes are important to you? [Or – Share more about that with me.]
- With this kind of therapy, is there anything you would be worried or concerned about?
- (If needed) Can you describe challenges or barriers you think your child might face in doing CIMT?
- (If asked/needed) Example barriers include: financial barriers, insurance barriers, time, and access to transportation.

**At-Home CIMT Questions**

Our guess is that some families may have difficulty making it to in-person therapy sessions as a part of CIMT. Our goal is to develop an at-home and caregiver-directed therapy program that families can confidently use if they cannot make it to in-person sessions, all while the child has their stronger arm casted.

- What are your thoughts on doing CIMT this way?
- Would wearing the cast for 24 hours for 3 weeks be feasible?
- What would be hard about a home-based CIMT approach?
- When developing this program, we want to explore child-caregiver interactions at home. Can you tell me about some of the games/activities you play with your child?
- Are these activities usually 1-on-1 or do they involve other family members?
- How often do you engage your child in these kinds of activities? (Explore time amounts for 1-on-1, group, and integrated activities)
- Describe what you think would be feasible for you to do with your child on a day-to-day basis with regard to time.
- At maximum, for a 3-week period, how much time per day would you be able to spend engaging in 1-on-1 activities with your child every day?
- (If needed) Can you describe how you came to this time estimate?
- If you were to engage in at-home therapies directed by you, how would you prefer to be trained on how to do those therapies?
- (If asked/needed) Examples include: attending an in-person or Zoom therapy session where a therapist shows you how to do the activities, detailed written explanations, a short overview sheet listing the activities, short videos demonstrating the activities, or a combination.
- Why would this be your preferred method of training?
- What concerns do you have about leading at-home therapies with your child?
- What advantages do you see in leading at-home therapies with your child?

**Activity Logging**

We would like people who follow an at-home therapy program to log the activities they do with their child every day.

- How would you prefer to log these daily activities?
- (If asked/needed) Examples include: a daily survey emailed to you, a written log sheet, or a smart-phone app.
- Why would this be your preferred logging method?
- Do you foresee any barriers to keeping a daily log of these activities?

**Interest in New Program**

Now, I’d like to learn more about your level of interest in doing CIMT and your goals for it.

- Would you be interested in pursuing CIMT with some at-home component for your child at this time? Why or why not?
- If you could design an ideal CIMT program for your child and family, what would it look like?
- How long would it be?
- What would the constraint/restraint part be like?
- How often would you want in-person components?
- (If asked/needed) Examples include: a therapist comes to your home and guides all therapies, you are trained on how to direct home-based therapies, a mix of in-person and home-based therapies, etc.
- What do you think are some benefits to having an at-home component of CIMT for you, your child, and/or your family? What are the challenges?
- What would be important to you to see in a CIMT program?
- (If asked/needed) Examples include: a therapist specialized in CIMT and hemiplegia; time in person vs. time at home, cost, availability of specific toys for CIMT, connection to community/region.
- Is there anything that would make you not want to participate in a home-based CIMT program?
- What would be the most important thing for you to know prior to starting a CIMT program here at St. Louis Children’s Hospital?
- (If asked/needed) Examples include: time commitment, experienced therapists, awareness of scope of CIMT and motor changes, continuous process of learning.

**Family Questions**

Now, I’d like to end by learning more about [name, your child with cerebral palsy] and your family.

- Tell me about [name, your child with cerebral palsy]
- (If asked/needed) What are their likes/dislikes? Describe their personality.
- Tell me about your family
- (If asked/needed) Who all lives at home? What do the adults do? Tell me about the kids in the home (schooling/personality/etc.)
- What does a typical day for your family look like?
- What does a typical day for [name, your child with cerebral palsy] look like?
- What kinds of therapies does [name, your child with cerebral palsy] currently engage in?
- What are your expectations for those therapies? What sorts of changes are you hoping to see?
- Are these therapies meeting your expectations?
- Have you been part of any parent or caregiver groups, or have you gotten to know any families who you feel have similar life experiences to yours? Can you share more about that with me?
- Is there anything else you’d like to share with us about [name, your child with cerebral palsy] or your family?

**Wrap Up Questions**

We are planning on offering CIMT that includes at-home therapies to a small group of our Cerebral Palsy Center families.

- Would you like to learn more about this program?
- Is there anything else you would like to share with me?

Thank you for sharing your experiences and expertise with us. Our goal is to use your responses to help craft an effective and accessible CIMT program for our Cerebral Palsy Center families.

Have a nice day!
